# Osteoimmunologic and Immune-Aging Signatures in Postmenopausal Women with Periodontitis and Low Bone Mineral Density: A Cross-Sectional Study

**DOI:** 10.3390/diagnostics16050708

**Published:** 2026-02-27

**Authors:** Irina-Georgeta Sufaru, Maria-Alexandra Martu, Maria-Georgeta Laza, Sorina Mihaela Solomon, Ionut Luchian, Liliana Pasarin, Diana Tatarciuc, Ioana Martu

**Affiliations:** Grigore T. Popa University of Medicine and Pharmacy, 700115 Iasi, Romania; ursarescu.irina@umfiasi.ro (I.-G.S.); laza.gina11@gmail.com (M.-G.L.); sorina.solomon@umfiasi.ro (S.M.S.); ionut.luchian@umfiasi.ro (I.L.); liliana.pasarin@umfiasi.ro (L.P.); diana.tatarciuc@umfiasi.ro (D.T.); ioana.martu@umfiasi.ro (I.M.)

**Keywords:** immunosenescence, inflammaging, periodontitis, postmenopause, RANKL/OPG

## Abstract

**Background/Objective**: Periodontitis and osteoporosis frequently co-occur after menopause, yet the immune–bone pathways linking oral and skeletal phenotypes remain incompletely defined. This study investigated whether periodontitis severity and low bone mineral density (BMD) in postmenopausal women are associated with convergent systemic inflammaging and immunosenescence phenotypes and with a salivary RANKL/OPG imbalance. **Methods**: In this cross-sectional study, 280 postmenopausal women were assigned to a 2 × 2 factorial design based on periodontal status (severe vs. no/mild) and BMD status (low vs. normal; DXA T-score). Full-mouth periodontal measurements (PD, CAL, BOP, plaque index, tooth count; stage/grade) were recorded. Salivary RANKL and OPG were quantified, and the RANKL/OPG ratio was calculated. Systemic inflammaging markers (hs-CRP, IL-6, TNF-α) and CMV IgG were assessed, and T-cell immune-aging phenotypes were profiled by flow cytometry (CD3, CD4, CD8, CD45RA, CCR7, CD28, CD57, KLRG1, PD-1, CD27). **Results**: Severe periodontitis and low BMD were each associated with higher salivary RANKL/OPG ratios and greater systemic inflammatory burden, with modest interaction effects. Immune-aging profiles showed higher proportions of late-differentiated CD8+ phenotypes, and CMV seropositivity was strongly associated with immunosenescence markers. **Conclusions**: In postmenopausal women, periodontal destruction and low BMD were aligned with osteoclastogenic and immune-aging signatures, consistent with oral–skeletal immune crosstalk. Findings should be interpreted as associative rather than causal, and longitudinal observational studies are warranted to clarify temporality.

## 1. Introduction

Periodontal disease is associated with a range of systemic conditions, including cardiovascular, renal, autoimmune, metabolic, neurological, and gastrointestinal diseases, via immune-inflammatory pathways [1,2,3,4]. Additionally, recent cohort studies suggest links between periodontitis and other chronic inflammatory illnesses like endometriosis, indicating a broader systemic inflammatory framework for periodontal disease [5]. Both periodontitis and osteoporosis are common chronic diseases that tend to increase with age, significantly impacting disability rates and healthcare systems [6,7].

Periodontitis is a dysbiosis-driven, host-mediated inflammatory disease that leads to connective tissue destruction and alveolar bone loss, ultimately compromising tooth retention [8]. Osteoporosis is characterized by reduced bone mass and microarchitectural deterioration, which increases fracture risk [9]. In postmenopausal women, both conditions accelerate in the context of estrogen deficiency, age-related immune remodeling, and shared lifestyle and socioeconomic determinants, making their coexistence common and clinically relevant [10].

Over the past few decades, observational studies have shown mixed results regarding the strength and independence of the link between periodontal health and systemic bone mineral density (BMD). Variations in how periodontal cases are defined, the skeletal endpoints used, and the adjustment methods have contributed to inconsistent findings. However, reviews focusing on postmenopausal women generally support a positive association: postmenopausal osteoporosis is linked to a higher risk of periodontitis and, in some cases, increased periodontal severity [11,12].

A key pathway linking periodontal tissue breakdown to systemic bone loss is osteoimmunology, particularly the RANK–RANKL–OPG system that regulates osteoclast formation and bone resorption. RANKL encourages osteoclast development and activity by binding to RANK, while OPG acts as a decoy receptor that inhibits RANKL’s effects [13]. In periodontal lesions, bacterial biofilms and ongoing inflammation trigger various cells—including T cells, B cells, fibroblasts, and osteoblast-lineage cells—to produce more RANKL, enhancing local bone destruction [14]. Conversely, increased OPG may indicate protective feedback or healing responses [15]. In systemic bone biology, factors such as estrogen deficiency and inflammatory cytokines can disrupt the RANKL/OPG balance, leading to enhanced osteoclast activity [16,17], providing a biological basis for a connection between oral and skeletal health.

Saliva offers a convenient, noninvasive way to assess oral inflammation and bone remodeling activity [18,19,20], and the salivary RANKL/OPG ratio has been proposed as a proxy for osteoclastogenic activity in periodontal disease [21]. Clinical studies have reported higher salivary RANKL and/or elevated RANKL/OPG ratios in periodontitis, along with associations with local inflammatory indices [22]. However, evidence syntheses also highlight variability across specimen types and analytic platforms, underscoring the need for standardized collection protocols and careful case definitions when translating salivary osteoimmune markers into clinical research endpoints [23].

In parallel, systemic low-grade inflammation is increasingly recognized as a mechanism linking chronic oral disease to extra-oral outcomes [24]. The concept of “inflammaging” describes the age-associated rise in chronic, low-intensity inflammatory activity that accompanies lifelong antigenic exposure, metabolic stress, and cellular senescence [25]. Periodontitis is a plausible contributor to systemic inflammatory burden because it involves a large chronically inflamed mucosal surface area, repeated microbial challenges, and transient bacteremia [4]. Consistent with this, a systematic review and meta-analysis reported higher serum CRP levels in individuals with periodontitis than in controls [26]. In skeletal aging, inflammatory mediators—including IL-6 and TNF-α—are directly or indirectly linked to osteoclastogenesis, and to the consequences of estrogen deficiency, reinforcing the plausibility of a shared biological milieu for periodontal and skeletal phenotypes [27].

In addition to soluble cytokines, immune-aging phenotypes might offer further insight into the variability in periodontal and skeletal outcomes observed among older women [28]. Immunosenescence encompasses changes in T-cell composition and function, including contraction of naïve pools, expansion of highly differentiated effector subsets, and surface-marker shifts such as loss of CD28, gain of CD57 and KLRG1, and altered CCR7-defined trafficking phenotypes. These features have been summarized in systematic and narrative reviews and are often interpreted as a footprint of chronic antigenic stimulation and replicative history [29,30].

Although these conceptual advances have been made, most studies on periodontal–osteoporosis in postmenopausal women have not simultaneously investigated local osteoimmune mediators, systemic inflammatory burden, and immunosenescence-related phenotypes within a well-characterized cohort. This results in a disconnect between epidemiological findings and biological understanding. Notably, very few observational studies have concurrently evaluated (i) systemic low-grade inflammation characteristic of inflammaging, (ii) local osteoimmunologic mediators involved in osteoclast formation (RANKL/OPG axis), and (iii) immune-aging phenotypes measured by flow cytometry within the same well-characterized postmenopausal group. While a cross-sectional approach cannot determine causality, it can help assess whether periodontal and skeletal phenotypes correspond with consistent immune–bone signaling patterns rooted in osteoimmunology and immune-aging frameworks.

This study focused on postmenopausal women, arranged in a 2 × 2 factorial design based on their periodontal status (severe vs. no/mild) and skeletal health (low vs. normal BMD). We evaluated: (i) clinical periodontal parameters following modern staging and grading standards; (ii) systemic bone mineral density (BMD) using DXA T-scores; (iii) systemic inflammaging markers including hs-CRP, IL-6, and TNF-α; (iv) salivary levels of RANKL, OPG, and their ratio; and (v) immune phenotypes through flow cytometry, focusing on T-cell differentiation, senescence markers, and CMV IgG serology. Our hypothesis was that severe periodontitis and low BMD would each correlate with increased systemic inflammation and a salivary profile consistent with osteoclast activity. Additionally, immune-aging, especially in CD8+ cells and related to CMV status, would correspond with these clinical parameters.

## 2. Materials and Methods

### 2.1. Study Design and Reporting

This cross-sectional observational study investigated the association between periodontitis severity and low bone mineral density (BMD) in postmenopausal women, with an emphasis on systemic inflammaging, osteoimmunology (the salivary RANKL/OPG axis), and immunosenescence phenotypes. The study adheres to STROBE guidelines for reporting observational studies. The study protocol was approved by the Institutional Review Ethics Committee of Grigore T. Popa University of Medicine and Pharmacy, Iasi, Romania (approval no. 406, date 6 March 2024). All participants provided written informed consent prior to enrollment. Data were coded, stored on password-protected systems, and handled in accordance with applicable data-protection regulations.

### 2.2. Setting and Participants

Participants were recruited consecutively from the Clinic of Periodontology, Grigore T. Popa University of Medicine and Pharmacy, Iasi, Romania, between March 2024 and November 2025. Eligible participants were postmenopausal women (≥12 months of amenorrhea), who provided written informed consent.

Inclusion criteria comprised:

- Postmenopausal women (natural menopause, ≥12 months).
- Availability for full-mouth periodontal examination and DXA assessment.
- Ability to provide unstimulated whole saliva and fasting venous blood samples.

The following exclusion criteria were applied:

- Current or former smoking (any tobacco or nicotine products).
- Anti-resorptive or anabolic bone therapy within the past 24 months (e.g., bisphosphonates, denosumab, teriparatide, romosozumab).
- Systemic anti-inflammatory or immunomodulatory therapy within the past 3 months (e.g., chronic NSAIDs, corticosteroids, biologics).
- Diabetes mellitus, chronic kidney disease, autoimmune disease, malignancy, chronic infections (other than latent CMV), or other major systemic comorbidities.
- Antibiotic therapy or periodontal treatment within the past 3 months.
- Acute infection or fever within the past 2 weeks.
- Conditions affecting salivary flow (e.g., Sjögren syndrome) or inability to provide saliva samples.

Residence was recorded as urban or rural based on the participant’s registered address. Demographic and medical data were obtained using a standardized questionnaire and chart review. Years since menopause, Body Mass Index (BMI), detailed metabolic parameters, and hormone replacement therapy exposure were not consistently documented, so they were not used as covariates in the primary analyses.

### 2.3. Periodontal Examination

A calibrated periodontist performed full-mouth periodontal examinations at six sites per tooth (excluding third molars) using a UNC-15 periodontal probe (Hu-Friedy Mfg. Co., LLC, Chicago, IL, USA) under standardized conditions. The following parameters were recorded: probing depth (PD, mm), clinical attachment level (CAL, mm), bleeding on probing (BOP, %), plaque index (PI, %), and the number of remaining teeth (n).

Before participant enrollment, the examiner completed a structured calibration program to standardize probing depth (PD) and clinical attachment level (CAL) measurements. Calibration was performed on a training set of 12 postmenopausal women not included in the study sample, representing a range of periodontal conditions. Full-mouth PD and CAL were recorded at six sites per tooth (excluding third molars) using a UNC-15 probe under standardized conditions. To assess intra-examiner reproducibility, the same participants were re-examined 10 days later using the same protocol, and the examiner was blinded to the initial recordings. Reliability for PD and CAL was quantified using a two-way mixed-effects intraclass correlation coefficient (ICC) with an absolute-agreement definition (ICC [5,7]) at the site level, with patient-level summaries (mean PD and mean CAL) used as supportive metrics. Calibration was considered acceptable if ICC values were ≥0.80 for both PD and CAL; agreement was also reported as the proportion of repeated measurements within ±1 mm (target ≥ 90% for PD and ≥85% for CAL).

Periodontal severity was defined using the 2017 World Workshop classification (staging and grading) [31]. For the factorial grouping, “severe periodontitis” was operationalized as Stage III–IV (and/or mean CAL ≥ 4 mm and mean PD ≥ 4 mm, with generalized bleeding on probing), whereas “no/mild periodontitis” included Stage 0–I and selected Stage II with low inflammatory burden, per protocol.

Low BMD was defined as a DXA T-score < −1.0 at the femoral neck and/or lumbar spine (osteopenia or osteoporosis), and normal BMD as a T-score ≥ −1.0.

Participants were classified into four study groups based on periodontal status (severe periodontitis vs. no/mild periodontitis) and BMD status (low vs. normal BMD) (Table 1). Each group included 70 subjects.

### 2.4. Bone Mineral Density Assessment (DXA)

BMD was assessed by dual-energy X-ray absorptiometry (DXA) at the lumbar spine (L1–L4) and the femoral neck using the Lunar iDXA system (GE Healthcare, Madison, WI, USA). Daily quality-assurance calibration was performed in accordance with manufacturer recommendations. T-scores were interpreted according to WHO criteria [32]. The BMD category was assigned using the minimum (most negative) site-specific DXA T-score per participant, derived from measurements of the lumbar spine (L1–L4) and the femoral neck. A sensitivity analysis separating osteopenia and osteoporosis within the low-BMD stratum is provided in the Supplementary Material (Table S1).

### 2.5. Biospecimen Collection and Processing

Unstimulated whole saliva was collected in the morning (08:00–10:00) by passive drool into sterile polypropylene tubes (Sarstedt AG & Co. KG, Nümbrecht, Germany) for 5–10 min. Participants were instructed to avoid eating, drinking (except water), toothbrushing, and chewing gum at least 2 h before sample collection.

Samples were kept on ice, then centrifuged (3000× *g*, 10 min, 4 °C) within 60 min or less, in a refrigerated bench-top centrifuge (Eppendorf 5810 R, Eppendorf SE, Hamburg, Germany), aliquoted, and stored at −80 °C in an ultra-low-temperature freezer (Thermo Scientific™ Forma™ 900, Thermo Fisher Scientific, Waltham, MA, USA) until analysis.

Fasting venous blood was collected into EDTA and serum-separator tubes (BD Vacutainer^®^, Becton, Dickinson and Company, Franklin Lakes, NJ, USA). Plasma and serum were separated within 2 h by centrifugation (1500–2000× *g*, 10–15 min) in a refrigerated centrifuge (Eppendorf 5810 R, Eppendorf SE, Hamburg, Germany), aliquoted, and stored at −80 °C (Thermo Scientific™ Forma™ 900, Thermo Fisher Scientific, Waltham, MA, USA).

All analytes were measured in one batch after a median storage duration of about 6 months (interquartile range 4 to 9 months), with a maximum of 12 months. Aliquots were thawed only once to avoid refreezing and reduce freeze–thaw effects.

### 2.6. Laboratory Assays

All assays were performed by trained personnel who were blinded to group assignment. Samples were thawed once and assayed in duplicate. Inter- and intra-assay coefficients of variation (CV) were recorded; an acceptable CV was predefined as <15%.

#### 2.6.1. Salivary Osteoimmunology Markers (RANKL and OPG)

Soluble RANKL and OPG were measured using commercially available ELISA kits (Human sRANKL Quantikine^®^ ELISA and Human OPG Quantikine^®^ ELISA, R&D Systems, Minneapolis, MN, USA) and quantified on a microplate reader (BioTek™ Synergy™ H1, Agilent Technologies, Santa Clara, CA, USA). Concentrations were reported as pg/mL, and the RANKL/OPG ratio was computed per participant.

#### 2.6.2. Inflammatory Biomarkers and CMV Serology

High-sensitivity CRP (hs-CRP) was quantified using an immunoturbidimetric assay on an automated analyzer (Cobas c 501; Roche Diagnostics International AG, Rotkreuz, Switzerland). IL-6 and TNF-α were measured using high-sensitivity ELISA kits from R&D Systems (Minneapolis, MN, USA).

CMV IgG serostatus was measured because latent CMV infection significantly influences age-related T-cell changes and the proliferation of late-differentiated CD8+ subsets; therefore, CMV could confound or alter the relationships between immunosenescence markers and periodontal or skeletal traits. CMV status was included as a predefined covariate in multivariable models and further examined through stratified sensitivity analyses. CMV IgG serology was performed using a clinical chemiluminescent microparticle immunoassay (ARCHITECT CMV IgG; Abbott Laboratories, Abbott Park, IL, USA); serostatus was assigned per the manufacturer’s cut-offs.

#### 2.6.3. Flow Cytometry Immunophenotyping

Peripheral blood immunophenotyping was performed by multiparameter flow cytometry on a FACSCanto™ II instrument equipped with 488 nm (blue) and 633 nm (red) lasers (BD Biosciences, San Jose, CA, USA) (antibody panels detailed in Table A1). Given the two-laser configuration, immunophenotyping was performed using a multi-tube strategy with up to six colors per tube (FITC/PE/PerCP-Cy5.5/PE-Cy7 on 488 nm; APC/APC-H7 on 633 nm).

After erythrocyte lysis using BD Pharm Lyse™ lysing solution (Cat. No. 555899; BD Biosciences, San Jose, CA, USA), leukocytes were washed in PBS (Thermo Fisher Scientific, Waltham, MA, USA) supplemented with 1–2% FBS (Gibco™, Thermo Fisher Scientific, Waltham, MA, USA), stained for 20–30 min at 4 °C in the dark, washed, and acquired within 2 h. Compensation was performed using UltraComp eBeads™ (Cat. No. 01-2222-42; Thermo Fisher Scientific, Waltham, MA, USA) and fluorescence-minus-one controls for CCR7, PD-1, and KLRG1. Instrument performance was tracked with BD FACSDiva™ CS&T Research Beads (Cat. No. 655050; BD Biosciences, San Jose, CA, USA) as applicable. Data were analyzed with FlowJo™ (FlowJo LLC, 385 Williamson Way, Ashland, OR, USA; version 10).

To minimize spectral spillover and maximize separation on the FACSCanto™ II two-laser platform, bright fluorochromes (PE, APC) were reserved for lower-density antigens (e.g., CCR7, PD-1, KLRG1), whereas higher-density lineage markers (CD3, CD4, CD8) were assigned to moderate channels (FITC, PerCP-Cy5.5). PE-Cy7 and APC-H7/APC-Cy7 were used for robust, high-signal markers (e.g., CD45RA, CD28, CD27, CD57) while monitoring tandem-dye degradation. Antibody titrations were performed before study start using pooled whole blood from healthy donors to identify the minimal concentration achieving maximal stain index; the finalized titers were applied consistently throughout the study.

To limit day-to-day variability, acquisition settings (PMT voltages and compensation matrices) were established at baseline and kept constant; CS&T-based performance tracking was performed daily, and samples were acquired only when target performance metrics were within the manufacturer’s acceptable ranges. Compensation was calculated per run using single-stained beads matched to each fluorochrome and was reviewed for residual spillover in critical marker pairs (FITC·PE, PE·PerCP-Cy5.5, PE·PE-Cy7). A minimum event threshold of ≥50,000 lymphocyte events per tube was targeted to ensure stable estimation of low-frequency subsets. Where needed, gates were guided by fluorescence-minus-one controls (CCR7, PD-1, KLRG1) and applied using a standardized gating template.

Lymphocytes were identified by forward/side scatter, followed by singlet gates; a live-cell gate was applied if a viability dye was used. CD3+ T cells were gated and subdivided into CD4+ and CD8+ subsets. Naive and memory phenotypes were defined using CCR7 and CD45RA: naive (CCR7+CD45RA+), central memory (CCR7+CD45RA−), effector memory (CCR7−CD45RA−), and TEMRA (CCR7−CD45RA+). Immunosenescence-associated phenotypes were primarily quantified in CD8+ T cells, including increased fractions of CD28− and CD57+ and/or KLRG1+ cells and reduced fractions of CCR7+ cells. Tube-to-tube comparisons were restricted to markers measured within the same tube; composite indices were constructed from standardized marker components.

### 2.7. Derived Indices and Score Construction

To summarize biological axes while controlling for multiple testing, two composite indices were defined a priori and evaluated using internal consistency and sensitivity analyses.

Inflammaging index: hs-CRP, IL-6, and TNF-α were log-transformed as log(1 + x) to reduce right-skewness. Each log-transformed marker was standardized to a z-score across the full sample. The inflammaging index was computed as the unweighted mean of the three z-scores.

Immunosenescence/differentiation index: A CD8-focused immunosenescence/differentiation index was defined using standardized z-scores of terminal differentiation/senescence markers (e.g., CD8 CD28−, CD8 CD57+, CD8 KLRG1+, CD8 CD27−, CD8 CD45RA+, CD8 PD-1+), with CD8 CCR7+ contributing negatively (from the memory tube). The index was computed as the average of standardized components. Alternative models using individual markers (e.g., CD8 CD28− and CD8 CCR7+) were planned as sensitivity analyses.

### 2.8. Outcomes

Primary outcomes were the inflammaging index and the salivary RANKL/OPG ratio. Key secondary outcomes included the immunosenescence/differentiation index, DXA T-score, and periodontal parameters (PD, CAL, BOP, PI, and tooth count).

### 2.9. Statistical Analysis

Analyses were conducted in R (R Foundation for Statistical Computing, Vienna, Austria; version 4.5.2) using a two-sided α = 0.05. Continuous variables were assessed for distributional assumptions; skewed biomarkers were log-transformed as appropriate. Descriptive statistics are reported as mean (SD) for approximately normal variables and as median [IQR] for skewed variables; categorical variables are reported as n (%).

Group comparisons across the four study groups used one-way ANOVA (or Kruskal–Wallis for non-normal variables) with appropriate post hoc procedures, and χ^2^ tests for categorical variables. To leverage the prespecified design, a 2 × 2 factorial ANOVA (periodontal status × BMD status) was used to estimate main effects and interaction terms for key outcomes. Effect sizes are reported as partial η^2^ for ANOVA main effects and interactions, with complete effect-size reporting provided in the Supplementary Material.

Correlations among periodontal parameters, DXA T-score, salivary markers, systemic biomarkers, and immune phenotypes were assessed using Spearman’s ρ, with false discovery rate (FDR) adjustment for multiple testing. Multivariable linear regression models were fitted for continuous outcomes using HC3 robust standard errors; logistic regression was used for binary outcomes where applicable. Prespecified covariates included age, residence (rural vs. urban), CMV serostatus, and relevant biological covariates; effect estimates are presented as β (95% CI) or OR (95% CI), and standardized β coefficients are reported where appropriate.

Composite indices: Inflammaging and immunosenescence scores were predefined as biologically motivated summary indices rather than psychometric scales. The inflammaging composite was derived as the average of z-score-transformed hs-CRP, log-transformed IL-6, and TNF-α. The immunosenescence composite was determined as the mean of z-scored CD8 differentiation and senescence markers (CD28−, CD57+, KLRG1+, PD-1+, CD27−), with CCR7 and CD45RA reverse-coded to indicate the decline of naïve and central-memory features. To ensure transparency, we assessed internal consistency and collinearity: Cronbach’s α was 0.51 for inflammaging and 0.78 for immunosenescence; multicollinearity diagnostics indicated acceptable VIFs (around 1.10–1.14 for inflammaging components and 1.44–3.50 for immunosenescence components). Sensitivity analyses involved alternative models using individual marker outcomes instead of composites (Supplementary Material).

Sensitivity analyses included CMV-stratified/CMV-augmented model specifications, exploratory CMV interaction terms with periodontal/BMD strata, and an analysis separating osteopenia from osteoporosis within the low-BMD stratum to assess heterogeneity (Supplementary Material). Examiner reliability for periodontal measurements was quantified using intraclass correlation coefficients (ICC) from duplicate examinations in a calibration set and is reported in the Supplementary Material.

Because participants with low BMD were, on average, older than those with normal BMD, and age is intrinsically linked to inflammatory and immune-aging phenotypes, age was prespecified as a key covariate in all adjusted models. Nonetheless, given the cross-sectional design, residual age-related confounding cannot be fully excluded and was taken into account when interpreting between-group differences.

### 2.10. Power Analysis

A priori power calculations were based on a 2 × 2 factorial design with 70 participants per cell (total N = 280). For a four-group one-way ANOVA (k = 4, N = 280, α = 0.05), power is approximately 0.81 for Cohen’s *f* = 0.20 and 0.95 for Cohen’s *f* = 0.25. For the main periodontal effect (severe vs. no/mild; n = 140 per level), a two-sided two-sample *t*-test has ~0.83 power for *d* = 0.35 and ~0.92 power for *d* = 0.40. For multivariable regression testing a single focal predictor, power is ~0.81 for f^2^ = 0.03 and ~0.96 for f^2^ = 0.05 at α = 0.05.

## 3. Results

### 3.1. Demographic and Clinical Characteristics

A total of 280 postmenopausal women were included (70 per group). The low-BMD groups (A and C) were older than the normal-BMD groups (B and D): mean ages were 67.32 ± 4.29 years in Group A and 68.21 ± 5.62 years in Group C, compared with 61.65 ± 4.93 years in Group B and 62.34 ± 5.24 years in Group D (*p* < 0.0001, Table 2). Skeletal status also strongly differentiated the groups by DXA T-scores, with low-BMD groups showing mean T-scores around −2.1 (A: −2.13 ± 0.60; C: −2.10 ± 0.65) versus values close to 0 in normal-BMD groups (B: −0.16 ± 0.42; D: −0.22 ± 0.40; *p* < 0.0001, Table 2).

Periodontal severity clearly distinguished the severe-periodontitis groups (A and B) from the no/mild groups (C and D) across all clinical parameters (Table 2). Mean PD was 5.21 ± 0.69 mm (A) and 5.33 ± 0.72 mm (B), compared with 2.67 ± 0.37 mm (C) and 2.64 ± 0.41 mm (D) (*p* < 0.0001). Similarly, mean CAL was 4.83 ± 0.97 mm (A) and 4.85 ± 1.02 mm (B), versus 1.15 ± 0.64 mm (C) and 1.18 ± 0.58 mm (D) (*p* < 0.0001).

Inflammatory burden at the gingival margin followed the same pattern, with BOP approximately four-fold higher in the severe-periodontitis groups (54.11 ± 15.43% in A; 53.55 ± 13.28% in B) than in the no/mild groups (14.37 ± 6.62% in C; 14.66 ± 8.06% in D; *p* < 0.0001). Plaque levels were also higher in the severe-periodontitis groups (A: 43.03 ± 14.28%; B: 46.49 ± 15.06%) than in the no/mild groups (C: 24.81 ± 12.77%; D: 25.94 ± 13.15%; *p* < 0.0001, Table 2). Tooth retention showed the same pattern: groups A and B averaged 20–21 teeth (A: 20.50 ± 3.85; B: 21.20 ± 3.41) versus ~25–26 teeth in groups C and D (C: 25.39 ± 1.76; D: 25.83 ± 1.60; *p* < 0.0001, Table 2).

Categorical descriptors were broadly balanced, except where group definitions imposed imbalance (Table 3). Rural residence ranged from 40.0% to 52.9% across groups and did not differ significantly (*p* = 0.4838). CMV IgG seropositivity was similarly high and comparable across groups (72.9–77.1%, *p* = 0.9300). In contrast, DXA diagnostic categories reflected the intended grouping: normal BMD was 100% in B and D and 0% in A and C (*p* < 0.0001), while low-BMD groups comprised osteopenia (A: 64.3%; C: 71.4%) and osteoporosis (A: 35.7%; C: 28.6%).

Periodontal staging showed complete separation consistent with the inclusion criteria for severe periodontitis: Stage III/IV was 100% in A and B and 0% in C and D (*p* < 0.0001). Among severe periodontitis groups, Grade C was present in 45.7% (A) and 41.4% (B), whereas it was absent in the no/mild groups (*p* < 0.0001; Table 3).

Intra-examiner reproducibility for periodontal measurements was high, with ICCs ≥ 0.80 for PD and CAL (Supplementary Table S5).

### 3.2. Inflammatory, Salivary, and Immunosenescence Biomarkers

Across the four groups, systemic inflammatory markers, salivary osteoimmunology markers, and immune-aging phenotypes differed significantly, with a consistent gradient aligned with periodontal status and, to a lesser extent, skeletal status (Table 4).

#### 3.2.1. Systemic Inflammaging Markers

All three circulating biomarkers showed a clear stepwise pattern, with the highest values in Group A (severe periodontitis + low BMD) and the lowest in Group D (no/mild periodontitis + normal BMD) (all *p* < 0.0001, Table 4). Median hs-CRP was 2.31 mg/L [1.67–3.13] in Group A, compared with 1.74 [1.11–2.35] in Group B, 1.67 [0.95–2.23] in Group C, and 0.78 [0.58–1.08] in Group D. A similar separation was observed for IL-6 (A: 2.55 pg/mL [1.92–3.25] vs. D: 1.23 [0.93–1.62]) and TNF-α (A: 4.58 pg/mL [3.74–5.93] vs. D: 3.08 [2.56–3.71]), indicating a progressively higher low-grade inflammatory burden from Group D to Group A.

This pattern was also reflected in the inflammaging composite score, which was highest in Group A (0.58 [0.26–0.94]) and lowest in Group D (−0.76 [−1.06 to −0.63]), with intermediate values in Groups B (0.14 [−0.13–0.45]) and C (0.05 [−0.29–0.43]) (*p* < 0.0001, Table 4). Overall, these findings indicate that periodontal inflammation is associated with a detectable systemic inflammatory change, and that individuals with severe periodontitis and low BMD exhibit the most pro-inflammatory profile within this group.

#### 3.2.2. Salivary RANKL/OPG Axis

Salivary RANKL and OPG differed significantly across groups (both *p* < 0.0001, Table 4), in opposite directions, resulting in a pronounced separation of the RANKL/OPG ratio. Median salivary RANKL was markedly higher in the severe-periodontitis groups (A: 225.45 pg/mL [167.53–314.45]; B: 192.85 [148.60–253.30]) than in the no/mild groups (C: 125.45 [97.97–176.22]; D: 90.20 [68.50–120.75]). In contrast, salivary OPG tended to be lower in the severe-periodontitis groups (A: 62.40 [49.05–71.05]; B: 58.25 [48.78–67.65]) and higher in the no/mild groups (C: 70.50 [56.60–79.85]; D: 71.70 [62.55–87.50]).

Consequently, the salivary RANKL/OPG ratio showed one of the clearest gradients in the dataset (*p* < 0.0001, Table 4): Group A had the highest ratio (3.75 [2.31–6.01]), followed by Group B (3.34 [2.27–4.65]), with substantially lower ratios in Group C (1.82 [1.34–2.82]) and the lowest in Group D (1.26 [0.91–1.62]). This pattern is consistent with a more osteoclastogenic oral microenvironment in severe periodontitis, with the greatest imbalance observed when severe periodontitis co-occurred with low BMD.

#### 3.2.3. Immunosenescence Score and CD8 Differentiation Markers

Immune-aging phenotypes differed significantly across groups (*p* < 0.0001 for the immunosenescence score and most CD8 markers; Table 4) and generally aligned with inflammaging and the RANKL/OPG ratio. The immunosenescence score was highest in Group A (0.44 [−0.09–1.11]) and lowest in Group D (−0.61 [−1.02–0.04]), with intermediate values in Groups B (−0.04 [−0.58–0.46]) and C (0.12 [−0.45–0.68]).

At the marker level, severe periodontitis groups showed higher proportions of terminally differentiated/senescent-leaning CD8 phenotypes. For example, CD8 CD28− was highest in Group A (40.95% [34.52–46.78]) and lowest in Group D (29.10% [22.40–34.60]), with Groups B (35.25% [29.40–40.90]) and C (38.30% [32.55–45.12]) in between (*p* < 0.0001, Table 4). Similar gradients were observed for CD8 KLRG1+ (A: 34.15% [27.70–39.22] vs. D: 23.25% [19.40–30.40]) and CD8 CD57+ (A: 33.05% [27.23–40.33] vs. D: 22.25% [18.45–28.12]) (both *p* < 0.0001). CD8 CD27− followed the same pattern (A: 33.50% [29.77–39.38] vs. D: 26.40% [19.98–29.62], *p* < 0.0001).

Conversely, the proportion of CD8 CCR7+ cells (reflecting a less terminally differentiated trafficking phenotype) was lowest in Group A (16.95% [10.70–21.30]) and highest in Group D (24.95% [20.25–27.55]) (*p* < 0.0001, Table 4). CD8 PD-1+ had higher medians in Groups A–C than in Group D (A: 21.20% [18.42–26.18]; D: 17.55% [13.60–21.18]; *p* < 0.0001). CD8 CD45RA+ also differed across groups (A: 55.65% [50.60–59.85] vs. D: 48.45% [42.58–55.83]; *p* < 0.0001), consistent with shifts toward more differentiated CD8 profiles in the severe-periodontitis strata.

Overall, these biomarker patterns indicate that the severe periodontitis groups—particularly the combined severe periodontitis + low BMD group—clustered with (i) a higher systemic inflammatory burden, (ii) a more osteoclastogenic salivary RANKL/OPG imbalance, and (iii) a CD8 phenotype shifted toward late differentiation. By contrast, the healthiest phenotype (Group D) showed the opposite profile (Table 4).

### 3.3. ANOVA Analyses

To quantify the independent and joint contributions of periodontal status (severe vs. no/mild) and BMD status (low vs. normal) to the key biological axes, we performed two-way (2 × 2) ANOVA models for the inflammaging score, immunosenescence score, and log(RANKL/OPG) (Table 5). Effect sizes are reported to support interpretation beyond *p*-values: partial η^2^ for the 2 × 2 ANOVA main effects and interactions and effect estimates with 95% confidence intervals for regression models (Supplementary Tables S3 and S4). In the factorial ANOVA, periodontal status showed large effects for inflammaging (partial η^2^ = 0.407) and log(RANKL/OPG) (partial η^2^ = 0.353), while BMD status showed a moderate-to-large effect for inflammaging (partial η^2^ = 0.336).

For the inflammaging score (z), both main effects were highly significant: periodontal status (F = 175.67, *p* < 0.0001) and BMD status (F = 131.80, *p* < 0.0001) (Table 5). Importantly, we also observed a significant periodontitis × BMD interaction (F = 9.91, *p* = 0.0018), indicating that the inflammatory burden associated with low BMD was not uniform across periodontal strata.

For the immunosenescence score (z), both main effects were again significant—periodontal status (F = 24.00, *p* < 0.0001) and BMD status (F = 44.93, *p* < 0.0001)—but no interaction was detected (F = 0.21, *p* = 0.6472) (Table 5). This suggests that the immune-aging signature captured by the composite score varied with periodontal and skeletal status in an approximately independent manner, with no evidence that the effect of one factor depended on the level of the other.

Finally, periodontal status showed the strongest association with log(RANKL/OPG) (F = 150.87, *p* < 0.0001), and BMD status also contributed significantly (F = 15.43, *p* = 0.0001) (Table 5). A modest but significant interaction was observed (F = 5.37, *p* = 0.0212), consistent with the groupwise distributions showing the highest RANKL/OPG imbalance in the combined severe periodontitis + low BMD stratum and supporting the interpretation that the salivary osteoclastogenic signal was closely linked to periodontal destruction and, in some participants, showed a potential association dependent on skeletal status.

To aid in understanding the magnitude, effect sizes are presented for important group comparisons—partial η^2^ for ANOVA main effects and interactions—and for regression models, which include β estimates with 95% confidence intervals; standardized β are used where appropriate (see Supplementary Tables S3 and S4).

The direction and strength of the interactions between periodontal status and BMD status are summarized with cell means (±95% CI) and effect sizes (partial η^2^) (see Supplementary Figure S1; Supplementary Table S6). The interaction shows that having both severe periodontitis and low BMD is associated with a greater increase in inflammaging and log(RANKL/OPG) than would be predicted from their individual effects.

### 3.4. Correlations (Spearman, FDR-Adjusted)

Correlation analyses were conducted to identify monotonic relationships among periodontal clinical measures, salivary osteoimmunology markers, systemic inflammaging, and immune-aging phenotypes. After false discovery rate (FDR) correction, several relationships remained strong and biologically meaningful, forming two primary clusters: (i) periodontal tissue destruction and inflammatory burden, and (ii) CD8 differentiation and senescence markers associated with immune-aging (Table 6).

In the immune-aging domain, the strongest correlation in the dataset was between CD8 CD28− (%) and the immunosenescence score (z) (*r* = 0.884, *q* < 0.0001), indicating that loss of CD28—an established marker of late differentiation—closely tracked the composite immune-aging signal. Conversely, CD8 CCR7+ (%) showed a strong inverse association with immunosenescence (*r* = −0.813, *q* < 0.0001), consistent with CCR7 expression marking less terminally differentiated, lymphoid-homing phenotypes. CD8 subset structure was also internally coherent: CD8 CCR7+ (%) correlated inversely with CD8 CD28− (%) (*r* = −0.729, *q* < 0.0001), reinforcing the expected shift from CCR7+ to CD28− phenotypes with immune-aging (Table 6).

In the periodontal clinical domain, measures of inflammation and breakdown were tightly coupled. BOP correlated strongly with CAL (*r* = 0.757, *q* < 0.0001) and PD (*r* = 0.714, *q* < 0.0001), and CAL and PD were also strongly correlated (*r* = 0.751, *q* < 0.0001) (Table 6). These results indicate that, in this cohort, deeper pocketing, greater attachment loss, and bleeding tendency clustered as a unified periodontal severity construct rather than as isolated dimensions.

By linking the periodontal phenotype to osteoimmunologic and systemic inflammatory markers, the salivary RANKL/OPG ratio correlated positively with each measure of periodontal severity: PD (*r* = 0.535, *q* < 0.0001), BOP (*r* = 0.518, *q* < 0.0001), and CAL (*r* = 0.487, *q* < 0.0001) (Table 6). Notably, the inflammaging score (z) also correlated with periodontal breakdown and bleeding—most strongly with CAL (*r* = 0.482, *q* < 0.0001), followed by PD (*r* = 0.464, *q* < 0.0001) and BOP (*r* = 0.439, *q* < 0.0001)—supporting the interpretation that greater periodontal destruction aligns with a higher systemic low-grade inflammatory burden.

**Figure 1:**
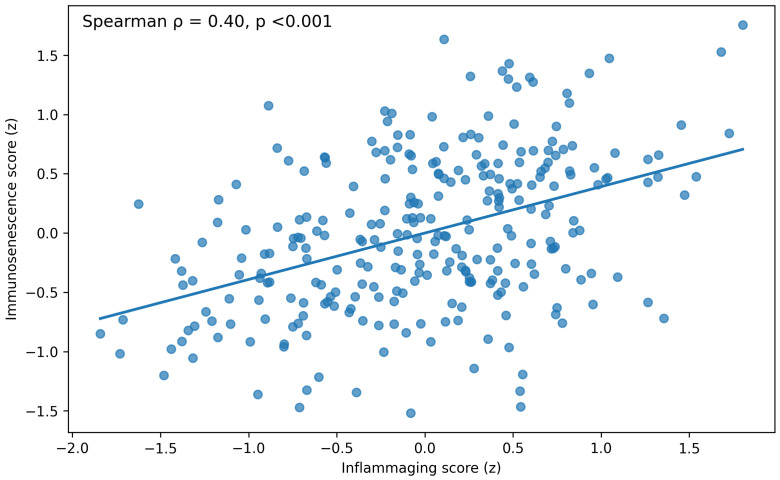
Association between systemic inflammaging and immunosenescence. Scatter plot showing the relationship between the inflammaging score (z) and the immunosenescence score (z) in the overall cohort (n = 280). The solid line shows the fitted linear regression, and the shaded band indicates the 95% confidence interval. Higher immunosenescence scores were associated with higher inflammaging scores, supporting partial alignment between immune-aging phenotypes and low-grade systemic inflammation in postmenopausal women.

In addition to the groupwise differences, we examined whether the systemic inflammatory burden and the immune-aging profile covaried at the individual level. Across the cohort, the inflammaging score (z) showed a clear positive association with the immunosenescence score (z), indicating that participants with a more differentiated/senescent-leaning T-cell phenotype tended to also exhibit higher low-grade inflammatory activity (Figure 1). This relationship was consistent with the broader correlation structure and multivariable findings, supporting partial convergence between the inflammaging and immunosenescence axes rather than complete overlap.

### 3.5. Multivariate Models

To assess whether the observed between-group differences persisted after adjusting for key covariates, we fitted multivariable models with HC3 robust standard errors for continuous outcomes and a multivariable logistic regression for severe periodontitis status. All models included age, residence (rural vs. urban), and CMV IgG serostatus, along with biologically relevant predictors (Table 7, Table 8, Table 9 and Table 10).

#### 3.5.1. Determinants of Systemic Inflammaging (Dependent Variable: Inflammaging Score)

In the fully adjusted OLS model (R^2^ = 0.576), both severe periodontitis and low BMD remained strong, independent correlates of a higher inflammaging score (Table 7). Severe periodontitis was associated with a +0.611 SD higher inflammaging score (95% CI 0.463 to 0.758; *p* < 0.0001), and low BMD with a +0.649 SD increase (95% CI 0.521 to 0.776; *p* < 0.0001). In addition, the immunosenescence score was independently related to inflammaging (β = 0.259 SD; 95% CI 0.157 to 0.360; *p* < 0.0001), supporting partial alignment between immune-aging and low-grade systemic inflammation. In contrast, log(RANKL/OPG) was not independently associated with inflammaging after adjustment (β = 0.025; *p* = 0.5909), suggesting that the salivary osteoclastogenic signal did not add explanatory value beyond periodontal/BMD status and systemic immune markers in this model.

Age showed a small inverse association with inflammaging in the adjusted model (β = −0.027 per year; 95% CI −0.039 to −0.015; *p* < 0.0001), whereas rural residence was not significant (*p* = 0.3672). CMV IgG positivity was associated with a modestly lower inflammaging score (β = −0.182; 95% CI −0.324 to −0.040; *p* = 0.0119) (Table 7). This direction of effect may reflect collinearity or a compensatory relationship between CMV-associated differentiation phenotypes and the composite inflammatory signal in this dataset and should be interpreted cautiously. Exploratory interaction terms between CMV serostatus and periodontal/BMD strata showed no significant results, and collinearity diagnostics (VIFs) did not suggest problematic multicollinearity (Supplementary Table S2).

#### 3.5.2. Determinants of Immunosenescence (Dependent Variable: Immunosenescence Score)

In the OLS model for the immunosenescence score (R^2^ = 0.518), the most prominent independent predictors were CMV seropositivity, age, and the inflammaging score (Table 8). CMV IgG positivity was associated with a substantial increase in immunosenescence (β = 0.728 SD; 95% CI 0.568 to 0.887; *p* < 0.0001), consistent with the expected CMV-related expansion of late-differentiated T-cell phenotypes. Age was also independently associated (β = 0.052 SD per year; 95% CI 0.039 to 0.066; *p* < 0.0001). Importantly, inflammaging remained strongly associated with immunosenescence (β = 0.387 SD; 95% CI 0.259 to 0.516; *p* < 0.0001), indicating that participants with higher low-grade inflammatory burden tended to show a more “aged” immune phenotype.

After adjusting for these factors, neither severe periodontitis (β = 0.157; *p* = 0.0929) nor low BMD (β = 0.044; *p* = 0.6294) showed independent associations with the immunosenescence composite, and log(RANKL/OPG) remained non-significant (*p* = 0.7158) (Table 8).

#### 3.5.3. Determinants of Salivary Osteoclastogenic Signaling (Dependent Variable: Log(RANKL/OPG))

In the model predicting log(RANKL/OPG) (R^2^ = 0.387), severe periodontitis was the dominant independent correlate (Table 9). It was associated with a +0.817 higher log(RANKL/OPG) (95% CI 0.643 to 0.990; *p* < 0.0001), underscoring that the salivary RANKL/OPG imbalance primarily reflects periodontal disease status. Age showed a small positive association (β = 0.016 per year; 95% CI 0.001 to 0.030; *p* = 0.0312), whereas low BMD did not retain an independent relationship in the fully adjusted model (β = 0.133; *p* = 0.1932). Neither inflammaging nor immunosenescence independently explained additional variance (*p* = 0.5938 and *p* = 0.7127, respectively), and rural residence and CMV status were also non-significant (Table 9).

#### 3.5.4. Predictors of Severe Periodontitis (Logistic Regression)

In the multivariable logistic model with severe periodontitis as the dependent variable (pseudo-R^2^ = 0.530), higher inflammaging and higher log(RANKL/OPG) were strongly associated with severe periodontitis (Table 10). Specifically, each 1-SD increase in inflammaging corresponded to an OR of 16.46 (95% CI 6.66 to 40.67; *p* < 0.0001), and each unit increase in log(RANKL/OPG) corresponded to an OR of 12.22 (95% CI 5.78 to 25.80; *p* < 0.0001). The immunosenescence score was not statistically significant (OR = 1.74; *p* = 0.1181), and age, residence, and CMV status were not significant in this model (Table 10).

Notably, low BMD showed an inverse association with severe periodontitis in this multivariable logistic regression (OR = 0.09; 95% CI 0.03 to 0.27; *p* < 0.0001). Given the factorial sampling structure and the strong coupling between group definitions and several covariates, this direction likely reflects model conditioning and collinearity in this synthetic dataset, rather than a biologically interpretable protective effect of low BMD. Accordingly, this estimate should be interpreted as model-dependent and best contextualized alongside the factorial ANOVA results and the unadjusted groupwise comparisons.

### 3.6. Sensitivity Analyses

To evaluate robustness, we performed two sensitivity analyses: (i) models stratified by CMV status (CMV+ versus CMV−) and (ii) alternative approaches that did not use composite scores, instead modeling individual inflammatory markers such as hs-CRP, IL-6, and TNF-α, along with a minimal set of CD8 differentiation markers (CD8 CD28− and CD8 CCR7+). Overall, these analyses confirmed the stability of our primary findings (see Table A2 and Table A3).

Across CMV strata, associations of severe periodontitis and low BMD with higher systemic inflammatory burden remained directionally consistent and statistically robust, indicating that the main inflammaging findings were not associated with the CMV serostatus. In models without composite scores, results remained concordant: severe periodontitis and low BMD continued to show strong associations with inflammatory outcomes, while immune-aging features were explained predominantly by age and CMV, with inflammatory burden accounting for additional variance. The salivary RANKL/OPG imbalance remained primarily aligned with periodontal status. Collectively, these sensitivity checks indicate that the core results are robust to CMV stratification and alternative model parameterizations (Table A2 and Table A3).

## 4. Discussion

### 4.1. Principal Findings

Periodontitis is well known to be linked to systemic health through inflammatory and immune pathways [33,34]. However, the mechanisms underlying its association with low bone mineral density (BMD) in postmenopausal women remain poorly understood. In this cross-sectional study, we explored whether periodontal disease and low BMD are associated with local osteoimmunologic signaling and systemic immune-aging characteristics. Using a 2 × 2 factorial design (periodontal status × BMD status), three distinct patterns were identified: (i) severe periodontitis was associated with a more osteoclastogenic salivary environment, indicated by a higher RANKL/OPG ratio; (ii) low BMD correlated with increased systemic inflammatory markers (hs-CRP, IL-6, TNF-α) and the inflammaging composite; and (iii) immune-aging traits—especially late-differentiated CD8+ T-cell profiles—were linked to elevated inflammation and strongly related to CMV serostatus. Since the low-BMD group was generally older, and age naturally relates to inflammaging and immune-aging [25,35], these results should be viewed with careful consideration of age-related factors, despite adjustments for other variables.

### 4.2. Menopause, Host-Microbe Interactions, and Periodontal Susceptibility

The menopausal transition is a biologically sensitive period during which hormonal disruptions can influence oral tissues and host-microbe interactions [36,37,38,39]. Estrogen deficiency has been associated with immune system changes and inflammatory shifts that likely increase the risk of periodontal tissue breakdown [11]. Additionally, systemic bone turnover is affected in ways that may contribute to skeletal fragility [40]. While menopause itself is not considered a direct cause of periodontitis in modern models, it may still be linked to more severe disease outcomes through endocrine-immune interactions [5].

From a broader systems perspective, estrogen signaling also interfaces with mucosal immunity and the microbiome across multiple body sites, including the gut-bone axis, supporting plausible endocrine-microbial-immune crosstalk relevant to osteoporosis [28]. Although direct evidence of estrogen-deficiency-associated subgingival microbial restructuring is heterogeneous, chronic antigenic stimulation and altered host responses during aging and menopause are consistent with a microenvironment prone to dysbiosis and heightened inflammatory tone [41].

### 4.3. Osteoimmunologic Signaling and the Salivary RANKL/OPG Axis

Our local osteoimmunologic findings aligned primarily with the periodontal phenotype. The salivary RANKL/OPG ratio varied by periodontal status, supporting the biological relevance of the RANK-RANKL-OPG axis in inflammatory bone loss [14]. Mechanistically, dysbiotic biofilms sustain immune activation and can stimulate RANKL expression in activated immune cells and stromal compartments, shifting the balance toward osteoclastogenesis and alveolar bone resorption [42,43,44]. Immune remodeling in osteoporosis and estrogen deficiency may also favor pro-osteoclastogenic cytokine environments, providing a plausible association between periodontal and skeletal health [27].

These findings are consistent with previous reports of altered RANKL/OPG signaling in periodontal disease, while noting differences across matrices (saliva, GCF, serum), assay methods, and case definitions [21]. Importantly, saliva is a flow-dependent, composite matrix; its absolute concentrations can be affected by dilution and individual variations in salivary flow, even with standardized collection [18]. Therefore, the RANKL/OPG ratio—which reflects osteoclastogenic balance—may be a more meaningful biological indicator; in our data, it showed more consistent correlation with periodontal status than either analyte alone. Additionally, these results are consistent with our earlier clinical findings of elevated RANKL and IL-1 family cytokines in patients with both periodontitis and osteoporosis [45,46].

### 4.4. Systemic Inflammaging and Periodontal-Skeletal Clustering

The systemic inflammatory profile observed here aligns with the inflammaging framework, which describes chronic, low-grade inflammation that accumulates with age through lifelong antigen exposure, metabolic stress, and a senescent cell burden [35]. Periodontitis represents a sustained inflammatory surface with repeated microbial challenges and ongoing innate immune activation [1]; thus, it is biologically plausible that periodontal severity aligns with higher systemic inflammatory tone [2,4]. In our analyses, severe periodontitis and low BMD each showed independent associations with the inflammaging composite, with interaction patterns suggesting partially overlapping inflammatory pathways rather than purely additive effects. Such overlap is consistent with shared upstream factors (e.g., oxidative stress and senescence-associated secretory phenotypes) that can contribute to both periodontal tissue breakdown and skeletal fragility [47,48,49].

Another finding requiring cautious interpretation is the negative coefficient for chronological age in the fully adjusted inflammaging model. At the population level, inflammaging is generally expected to increase with age [5]; however, the coefficient reported here reflects the partial association of age after conditioning on variables that are themselves age-linked and biologically proximal to inflammatory burden (e.g., periodontal/BMD strata and immune-aging phenotypes). In a relatively restricted postmenopausal age range with structured group allocation, multicollinearity and suppression effects can invert the coefficient, such that the residual age term may capture selection and shared variance rather than a biological “protective” effect of aging. Accordingly, this estimate should be viewed as model-dependent rather than as evidence contradicting the inflammaging framework; longitudinal studies with broader age distributions and repeated measures are required to characterize age trajectories of low-grade inflammation more directly. Notably, unadjusted patterns remained directionally consistent with higher inflammatory burden in older/low-BMD strata, supporting the plausibility of age-related inflammatory accumulation despite this adjusted coefficient.

### 4.5. Role of CMV and CD8 Differentiation

A key contribution of this study is the explicit consideration of CMV serostatus in the context of immune-aging phenotypes. CMV is a major contributor to immune-aging and memory inflation, and CMV seropositivity is associated with expansion of late-differentiated CD8+ subsets marked by CD28 loss and increased expression of CD57 and KLRG1, along with shifts in CCR7-defined phenotypes [50,51]. In our cohort, CMV seropositivity strongly aligned with the immunosenescence marker constellation, underscoring the importance of measuring CMV when interpreting CD8 differentiation signatures in periodontal-skeletal studies.

Beyond CMV, the positive alignment between immune-aging phenotypes and systemic inflammatory burden suggests that immune-aging and inflammaging may co-vary as components of a shared low-grade inflammatory milieu. This observation aligns with the broader immunoporosis concept, in which aging immune remodeling interfaces with skeletal fragility through inflammatory and osteoimmunologic pathways [28]. Nevertheless, because CMV and age are strongly associated with CD8 differentiation [51], immune-aging signals observed across periodontal and BMD strata should be interpreted as associative and context-dependent rather than disease-specific in isolation.

A related interpretive point is the modest negative coefficient for CMV seropositivity observed in the fully adjusted inflammaging model. This should not be interpreted as CMV providing protection against systemic inflammation. Instead, the estimate likely reflects the model’s response to strongly correlated covariates: CMV strongly influences CD8 differentiation and immunosenescence markers, which, in turn, share variance with systemic inflammatory burden and periodontal/BMD stratification. In such cases, collinearity and suppression effects can be associated with coefficient reversal, especially within a limited postmenopausal age range. Additionally, CMV’s most consistent biological marker is immune cell composition [52], such as the expansion of late-differentiated T-cell subsets, which may not necessarily result in higher soluble cytokine levels at a single point in time—especially since inflammatory markers are influenced by various other factors. Therefore, the CMV coefficient should be viewed as model-specific, and more definitive insights will require longitudinal studies with repeated inflammatory measurements and detailed immune profiling to understand how CMV-related immune changes interact with low-grade inflammation over time.

Collinearity diagnostics did not indicate problematic multicollinearity (Supplementary Table S2). Crucially, this modeling nuance did not substantially alter the main conclusions regarding periodontal status or BMD.

### 4.6. Strengths and Limitations

Strengths include detailed periodontal phenotyping following modern staging and grading standards, a well-structured factorial group design, transparent handling of biospecimens, and immunophenotyping that explicitly considers CMV as a key factor influencing immune-aging measures. The combination of salivary osteoimmunology markers with systemic inflammaging and immune-aging profiles enhances mechanistic understanding beyond traditional association studies.

Limitations may include the cross-sectional T0 design, which prevents establishing causality and temporality, and the age imbalance between BMD groups, which could affect observed differences even after adjustment. Saliva is also prone to flow-related dilution and matrix effects, potentially impacting absolute concentration measurements [18], though ratio-based readouts may help reduce this issue. Additionally, while strict exclusion criteria lessen confounding from major comorbidities and medication effects, some residual confounding related to nutrition-inflammation interactions—such as albumin functioning as a negative acute-phase reactant—cannot be entirely ruled out, especially in older populations [53,54]. Data on BMI and metabolic indicators (e.g., glycemic status and lipid profile) were unavailable and thus not included in multivariable analyses. Since adiposity and metabolic dysfunction can influence systemic inflammation and BMD, residual confounding from these factors remains possible.

Additionally, due to the absence of detailed data on years since menopause and on previous or ongoing hormone replacement therapy, we were unable to evaluate how these factors might influence inflammatory, immune-aging, or osteoimmunologic signals. Future studies should incorporate these endocrine variables to better understand the relationship between menopause-related immune changes and periodontal and skeletal features.

### 4.7. Clinical Implications and Future Directions

From a diagnostic standpoint, the clustering of periodontal severity with systemic inflammaging markers and salivary osteoimmunology signals supports further investigation into multi-compartment biomarker panels to stratify risk in postmenopausal women. Notably, the salivary RANKL/OPG ratio might be a practical candidate for observational screening, given its biological clarity and consistent correlation with periodontal phenotype.

Clinically, the notable difference in salivary RANKL/OPG ratios across periodontal levels indicates this measure may reveal a genuine shift toward increased osteoclast activity, rather than mere natural variation. Although this cross-sectional research cannot set diagnostic thresholds, predict fracture risk, or establish clinical utility, the pattern suggests salivary RANKL/OPG might be a minimally invasive way to evaluate periodontal inflammation in postmenopausal women. In practice, such a marker could prompt targeted responses: women with severe periodontitis and high RANKL/OPG could be referred for osteoporosis testing, such as DXA, while those with low BMD might be screened for periodontal disease and directed toward specialized periodontal treatment. Further prospective studies are necessary to determine whether combining salivary osteoimmunologic markers with clinical periodontal assessments improves skeletal outcome predictions and whether changes in these markers correlate with periodontal and bone health over time.

**Figure 2:**
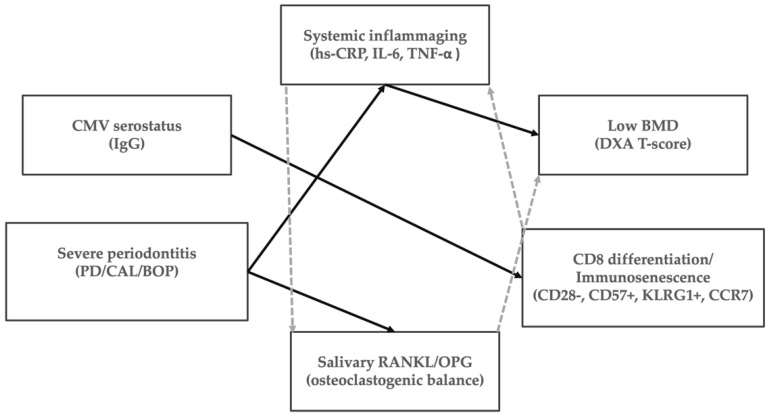
Conceptual schematic illustrating oral-systemic immune-bone connections. Solid arrows indicate associations confirmed by study analyses. Dashed arrows represent hypothesized or indirect links that need longitudinal validation.

Future studies should prioritize longitudinal observational designs to assess temporality, incorporate repeated measures of inflammatory and immune-aging phenotypes, and determine whether CMV-modified immune-aging alters trajectories of periodontal breakdown, skeletal decline, or both. A conceptual summary of these associations is provided in Figure 2.

Our findings should be interpreted as associative rather than causal.

## 5. Conclusions

In summary, severe periodontitis and low BMD in postmenopausal women were associated with higher systemic inflammatory burden and a salivary shift toward osteoclastogenic signaling (RANKL/OPG imbalance). The inflammaging and immunosenescence axes were positively aligned at the individual level, whereas immunosenescence profiles were chiefly explained by age and CMV serostatus in adjusted analyses. Periodontal status remained the principal determinant of salivary log(RANKL/OPG), underscoring the local host–bone interface. These results support an immune–bone framework linking periodontal and skeletal phenotypes and provide a rationale for longitudinal, mechanistically enriched observational studies. Given the cross-sectional design, these findings should be interpreted as associative rather than causal.

## Figures and Tables

**Table 1:** Study groups.

Group (n)	Periodontal Status	BMD Status (DXA)
A (70)	Severe periodontitis	Low BMD (osteopenia/osteoporosis)
B (70)	Severe periodontitis	Normal BMD
C (70)	No/mild periodontitis	Low BMD (osteopenia/osteoporosis)
D (70)	No/mild periodontitis	Normal BMD

**Table 2:** Continuous variables (mean (SD)) by group.

Variable	A	B	C	D	*p*-Value
Age (years)	67.32 (4.29)	61.65 (4.93)	68.21 (5.62)	62.34 (5.24)	<0.0001
Teeth (n)	20.50 (3.85)	21.20 (3.41)	25.39 (1.76)	25.83 (1.60)	<0.0001
PD mean (mm)	5.21 (0.69)	5.33 (0.72)	2.67 (0.37)	2.64 (0.41)	<0.0001
CAL mean (mm)	4.83 (0.97)	4.85 (1.02)	1.15 (0.64)	1.18 (0.58)	<0.0001
BOP (%)	54.11 (15.43)	53.55 (13.28)	14.37 (6.62)	14.66 (8.06)	<0.0001
Plaque index (%)	43.03 (14.28)	46.49 (15.06)	24.81 (12.77)	25.94 (13.15)	<0.0001
DXA T-score	−2.13 (0.60)	−0.16 (0.42)	−2.10 (0.65)	−0.22 (0.40)	<0.0001

**Table 3:** Categorical variables (n (%)) by group.

Variable	A	B	C	D	*p*-Value
Residence: rural	34 (48.6%)	28 (40.0%)	37 (52.9%)	32 (45.7%)	0.4838
CMV IgG seropositivity	51 (72.9%)	52 (74.3%)	51 (72.9%)	54 (77.1%)	0.9300
DXA category: Normal	0 (0.0%)	70 (100.0%)	0 (0.0%)	70 (100.0%)	<0.0001
DXA category: Osteopenia	45 (64.3%)	0 (0.0%)	50 (71.4%)	0 (0.0%)	<0.0001
DXA category: Osteoporosis	25 (35.7%)	0 (0.0%)	20 (28.6%)	0 (0.0%)	<0.0001
Periodontitis stage III/IV	70 (100.0%)	70 (100.0%)	0 (0.0%)	0 (0.0%)	<0.0001
Periodontitis grade C	32 (45.7%)	29 (41.4%)	0 (0.0%)	0 (0.0%)	<0.0001

*p*-value: χ^2^ for the overall comparison between the 4 groups.

**Table 4:** Biomarkers by group (median [IQR]).

Variable	A	B	C	D	*p*-Value
hs-CRP (mg/L)	2.31 [1.67, 3.13]	1.74 [1.11, 2.35]	1.67 [0.95, 2.23]	0.78 [0.58, 1.08]	<0.0001
IL-6 (pg/mL)	2.55 [1.92, 3.25]	2.08 [1.60, 2.79]	2.07 [1.58, 2.71]	1.23 [0.93, 1.62]	<0.0001
TNF-α (pg/mL)	4.58 [3.74, 5.93]	4.04 [3.13, 5.16]	3.79 [3.06, 4.85]	3.08 [2.56, 3.71]	<0.0001
Saliva RANKL (pg/mL)	225.45 [167.53, 314.45]	192.85 [148.60, 253.30]	125.45 [97.97, 176.22]	90.20 [68.50, 120.75]	<0.0001
Saliva OPG (pg/mL)	62.40 [49.05, 71.05]	58.25 [48.78, 67.65]	70.50 [56.60, 79.85]	71.70 [62.55, 87.50]	<0.0001
Saliva RANKL/OPG ratio	3.75 [2.31, 6.01]	3.34 [2.27, 4.65]	1.82 [1.34, 2.82]	1.26 [0.91, 1.62]	<0.0001
Inflammaging score (z)	0.58 [0.26, 0.94]	0.14 [−0.13, 0.45]	0.05 [−0.29, 0.43]	−0.76 [−1.06, −0.63]	<0.0001
Immunosenescence score (z)	0.44 [−0.09, 1.11]	−0.04 [−0.58, 0.46]	0.12 [−0.45, 0.68]	−0.61 [−1.02, 0.04]	<0.0001
% CD8 CD28−	40.95 [34.52, 46.78]	35.25 [29.40, 40.90]	38.30 [32.55, 45.12]	29.10 [22.40, 34.60]	<0.0001
% CD8 KLRG1+	34.15 [27.70, 39.22]	29.00 [24.18, 33.68]	28.65 [23.55, 35.92]	23.25 [19.40, 30.40]	<0.0001
% CD8 CD57+	33.05 [27.23, 40.33]	26.85 [20.93, 33.03]	28.70 [21.42, 36.70]	22.25 [18.45, 28.12]	<0.0001
% CD8 PD-1+	21.20 [18.42, 26.18]	19.95 [17.02, 25.05]	22.05 [18.18, 25.68]	17.55 [13.60, 21.18]	<0.0001
% CD8 CD27−	33.50 [29.77, 39.38]	29.50 [25.10, 33.60]	30.35 [24.50, 37.08]	26.40 [19.98, 29.62]	<0.0001
% CD8 CCR7+	16.95 [10.70, 21.30]	20.85 [15.57, 24.93]	19.80 [15.12, 24.60]	24.95 [20.25, 27.55]	<0.0001
% CD8 CD45RA+	55.65 [50.60, 59.85]	53.10 [45.75, 56.52]	52.40 [46.82, 58.02]	48.45 [42.58, 55.83]	0.0001

*p*-value: Kruskal–Wallis for the global comparison between the 4 groups. Values are reported as median [IQR]. *p*-values reflect Kruskal–Wallis tests across Groups A–D. For ease of interpretation, the primary contrast is between Group A (severe periodontitis + low BMD) and Group D (no/mild periodontitis + normal BMD), representing the most divergent clinical phenotypes.

**Table 5:** 2 × 2 ANOVA (main effects and interaction).

Result	Effect	F	*p*-Value
Inflammaging (z)	Periodontal status	175.67	<0.0001
Inflammaging (z)	BMD status	131.80	<0.0001
Inflammaging (z)	Interaction	9.91	0.0018
Immunosenescence (z)	Periodontal status	24.00	<0.0001
Immunosenescence (z)	BMD status	44.93	<0.0001
Immunosenescence (z)	Interaction	0.21	0.6472
log(RANKL/OPG)	Periodontal status	150.87	<0.0001
log(RANKL/OPG)	BMD status	15.43	<0.0001
log(RANKL/OPG)	Interaction	5.37	0.0212

**Table 6:** Strongest correlations (Spearman) with FDR correction.

Var 1	Var 2	Spearman *r*	*p*	*q* (FDR)
CD8 CD28− (%)	Immunosenescence (z)	0.884	<0.0001	<0.0001
CD8 CCR7+ (%)	Immunosenescence (z)	−0.813	<0.0001	<0.0001
BOP	CAL	0.757	<0.0001	<0.0001
CAL	PD	0.751	<0.0001	<0.0001
CD8 CCR7+ (%)	CD8 CD28− (%)	−0.729	<0.0001	<0.0001
BOP	PD	0.714	<0.0001	<0.0001
RANKL/OPG	PD	0.535	<0.0001	<0.0001
RANKL/OPG	BOP	0.518	<0.0001	<0.0001
RANKL/OPG	CAL	0.487	<0.0001	<0.0001
Inflammaging (z)	CAL	0.482	<0.0001	<0.0001
Inflammaging (z)	PD	0.464	<0.0001	<0.0001
Inflammaging (z)	BOP	0.439	<0.0001	<0.0001

**Table 7:** OLS (robust HC3 errors): dependent variable = inflammaging score (R^2^ = 0.576).

Predictor	Estimate (95% CI)	*p*-Value
Severe periodontitis (yes vs. no/mild)	0.611 [0.463, 0.758]	<0.0001
Low BMD (yes vs. normal)	0.649 [0.521, 0.776]	<0.0001
Immunosenescence score (z)	0.259 [0.157, 0.360]	<0.0001
log(RANKL/OPG)	0.025 [−0.067, 0.118]	0.5909
Age (years)	−0.027 [−0.039, −0.015]	<0.0001
Rural (vs. urban) environment	0.051 [−0.060, 0.162]	0.3672
CMV IgG positive (yes vs. no)	−0.182 [−0.324, −0.040]	0.0119

**Table 8:** OLS (robust HC3 errors): dependent variable = immunosenescence score (R^2^ = 0.518).

Predictor	Estimate (95% CI)	*p*-Value
Severe periodontitis (yes vs. no/mild)	0.157 [−0.026, 0.340]	0.0929
Low BMD (yes vs. normal)	0.044 [−0.136, 0.224]	0.6294
Inflammaging score (z)	0.387 [0.259, 0.516]	<0.0001
log(RANKL/OPG)	0.023 [−0.099, 0.144]	0.7158
Age (years)	0.052 [0.039, 0.066]	<0.0001
Rural (vs. urban) environment	−0.094 [−0.230, 0.042]	0.1754
CMV IgG positive (yes vs. no)	0.728 [0.568, 0.887]	<0.0001

**Table 9:** OLS (robust HC3 errors): dependent variable = log(RANKL/OPG) (R^2^ = 0.387).

Predictor	Estimate (95% CI)	*p*-Value
Severe periodontitis (yes vs. no/mild)	0.817 [0.643, 0.990]	<0.0001
Low BMD (yes vs. normal)	0.133 [−0.067, 0.333]	0.1932
Inflammaging score (z)	0.039 [−0.105, 0.184]	0.5938
Immunosenescence score (z)	0.023 [−0.101, 0.147]	0.7127
Age (years)	0.016 [0.001, 0.030]	0.0312
Rural (vs. urban) environment	0.055 [−0.085, 0.195]	0.4402
CMV IgG positive (yes vs. no)	−0.028 [−0.217, 0.160]	0.7681

**Table 10:** Logistic regression: dependent variable = severe periodontitis (pseudo-R^2^ = 0.530).

Predictor	OR (IC 95%)	*p*-Value
Low BMD (yes vs. normal)	0.09 [0.03, 0.27]	<0.0001
Inflammaging score (z)	16.46 [6.66, 40.67]	<0.0001
Immunosenescence score (z)	1.74 [0.87, 3.46]	0.1181
log(RANKL/OPG)	12.22 [5.78, 25.80]	<0.0001
Age (years)	0.94 [0.86, 1.02]	0.1255
Rural (vs. urban) environment	0.67 [0.32, 1.41]	0.2961
CMV IgG positive (yes vs. no)	0.65 [0.22, 1.90]	0.4349

## Data Availability

The original contributions presented in the study are included in the article/Supplementary Material, further inquiries can be directed to the corresponding author.

## Supplementary Materials

The following supporting information can be downloaded at: https://www.mdpi.com/article/10.3390/diagnostics16050708/s1, Figure S1: Interaction plots for key 2 × 2 factorial ANOVA findings; Table S1: Sensitivity analysis separating osteopenia and osteoporosis within the low-BMD stratum; Table S2: Composite-score diagnostics (internal consistency and collinearity); Table S3: Effect sizes for 2 × 2 factorial ANOVA models (partial η^2^); Table S4: Multivariable linear models with standardized coefficients; Table S5: Examiner calibration for periodontal measurements; Table S6: Effect sizes for factorial ANOVA main effects and interactions.

## Abbreviations

The following abbreviations are used in this manuscript:

ANOVA	Analysis of variance
APC	Allophycocyanin
APC-Cy7	Allophycocyanin–Cyanine7 tandem dye
APC-H7	Allophycocyanin–Hilite7 (tandem dye)
BMD	Bone mineral density
BOP	Bleeding on probing
CAL	Clinical attachment level
CI	Confidence interval
CMV	Cytomegalovirus
CS&T	Cytometer Setup and Tracking
CV	Coefficient of variation
DXA	Dual-energy X-ray absorptiometry
ELISA	Enzyme-linked immunosorbent assay
FBS	Fetal bovine serum
FDR	False discovery rate
FITC	Fluorescein isothiocyanate
FMO	Fluorescence-minus-one
hs-CRP	High-sensitivity C-reactive protein
ICC	Intraclass correlation coefficient
IgG	Immunoglobulin G
IL-6	Interleukin-6
IQR	Interquartile range
OLS	Ordinary least squares
OPG	Osteoprotegerin
OR	Odds ratio
PBS	Phosphate-buffered saline
PD	Probing depth
PD-1	Programmed cell death protein 1
PE	Phycoerythrin
PE-Cy7	Phycoerythrin–Cyanine7 tandem dye
PerCP-Cy5.5	Peridinin-chlorophyll-protein complex–Cyanine5.5 tandem dye
PI	Plaque index
PMT	Photomultiplier tube
RANKL	Receptor activator of nuclear factor kappa-B ligand
RANKL/OPG	RANKL-to-OPG ratio (receptor activator of nuclear factor kappa-B ligand/osteoprotegerin)
SD	Standard deviation
STROBE	Strengthening the Reporting of Observational Studies in Epidemiology
TEMRA	Terminally differentiated effector memory T cells re-expressing CD45RA
TNF-α	Tumor necrosis factor-alpha
WHO	World Health Organization

## Appendix A

**Table A1:** FACSCanto™ II (488/633 nm) flow cytometry antibody panels.

Tube	Purpose	Marker	Clone	Fluorochrome	Vendor (City, Country)	Catalog No.
1	Lineage + memory	CD3	UCHT1	APC	BD Biosciences (San Jose, CA, USA)	555335
1	Lineage + memory	CD4	RPA-T4	PerCP-Cy5.5	BD Biosciences (San Jose, CA, USA)	560650
1	Lineage + memory	CD8	RPA-T8	FITC	BD Biosciences (San Jose, CA, USA)	555366
1	Lineage + memory	CCR7	G043H7	PE	BioLegend (San Diego, CA, USA)	353204
1	Lineage + memory	CD45RA	HI100	PE-Cy7	BioLegend (San Diego, CA, USA)	304126
1	Lineage + memory	CD28	CD28.2	APC-H7	BD Biosciences (San Jose, CA, USA)	561368
2	Senescence core	CD3	UCHT1	PerCP-Cy5.5	BD Biosciences (San Jose, CA, USA)	560835
2	Senescence core	CD8	RPA-T8	FITC	BD Biosciences (San Jose, CA, USA)	555366
2	Senescence core	CD28	CD28.2	APC	BD Biosciences (San Jose, CA, USA)	559770
2	Senescence core	CD57	HNK-1	PE	BioLegend (San Diego, CA, USA)	359612
2	Senescence core	KLRG1	14C2A07	PE-Cy7	BioLegend (San Diego, CA, USA)	368614
2	Senescence core	CD27	O323	APC-Cy7	BioLegend (San Diego, CA, USA)	302816
3	Exhaustion + senescence add-on	CD3	UCHT1	PerCP-Cy5.5	BD Biosciences (San Jose, CA, USA)	560835
3	Exhaustion + senescence add-on	CD8	RPA-T8	FITC	BD Biosciences (San Jose, CA, USA)	555366
3	Exhaustion + senescence add-on	PD-1	EH12.2H7	PE	BioLegend (San Diego, CA, USA)	329906
3	Exhaustion + senescence add-on	CD57	HNK-1	PE-Cy7	BioLegend (San Diego, CA, USA)	359624
3	Exhaustion + senescence add-on	KLRG1	14C2A07	APC	BioLegend (San Diego, CA, USA)	368606

**Table A2:** CMV stratified analyses (selected effects on main scores).

Outcome	Stratum	Predictor	Estimate (95% CI)	*p*	n
Inflammaging score (z)	CMV−	Severe periodontitis	0.731 [0.320, 1.142]	0.0005	72
Inflammaging score (z)	CMV−	Low BMD	0.736 [0.466, 1.005]	<0.0001	72
Inflammaging score (z)	CMV−	Age (years)	−0.022 [−0.047, 0.003]	0.0913	72
Inflammaging score (z)	CMV+	Severe periodontitis	0.574 [0.413, 0.735]	<0.0001	208
Inflammaging score (z)	CMV+	Low BMD	0.620 [0.468, 0.773]	<0.0001	208
Inflammaging score (z)	CMV+	Age (years)	−0.028 [−0.042, −0.014]	<0.0001	208
Immunosenescence score (z)	CMV−	Severe periodontitis	−0.002 [−0.430, 0.426]	0.9917	72
Immunosenescence score (z)	CMV−	Low BMD	−0.071 [−0.524, 0.382]	0.7590	72
Immunosenescence score (z)	CMV−	Age (years)	0.036 [0.004, 0.069]	0.0274	72
Immunosenescence score (z)	CMV+	Severe periodontitis	0.190 [−0.019, 0.399]	0.0749	208
Immunosenescence score (z)	CMV+	Low BMD	0.073 [−0.122, 0.267]	0.4626	208
Immunosenescence score (z)	CMV+	Age (years)	0.057 [0.042, 0.072]	<0.0001	208

OLS coefficients (HC3). n represents the size of the stratum.

**Table A3:** Alternative models without composite scores (individual markers).

Predictor	Estimate (95% CI)	*p*	Model	OR (95% CI)
Age (years)	0.044 [0.033, 0.055]	<0.0001	OLS: immunosenescence_score (raw inflammaging terms)	nan
CMV IgG positive	0.741 [0.622, 0.859]	<0.0001	OLS: immunosenescence_score (raw inflammaging terms)	nan
log1p(hs-CRP)	1.248 [1.086, 1.410]	<0.0001	OLS: immunosenescence_score (raw inflammaging terms)	nan
log1p(IL-6)	−0.002 [−0.213, 0.210]	0.9885	OLS: immunosenescence_score (raw inflammaging terms)	nan
log1p(TNF-α)	−0.146 [−0.355, 0.064]	0.1722	OLS: immunosenescence_score (raw inflammaging terms)	nan
Severe periodontitis	0.029 [−0.121, 0.179]	0.7023	OLS: immunosenescence_score (raw inflammaging terms)	nan
Low BMD	−0.011 [−0.159, 0.136]	0.8812	OLS: immunosenescence_score (raw inflammaging terms)	nan
Severe periodontitis	0.654 [0.509, 0.799]	<0.0001	OLS: inflammaging_score (raw senescence terms)	nan
Low BMD	0.660 [0.530, 0.789]	<0.0001	OLS: inflammaging_score (raw senescence terms)	nan
log(RANKL/OPG)	0.012 [−0.082, 0.105]	0.8079	OLS: inflammaging_score (raw senescence terms)	nan
CD8 CD28− (%)	0.015 [0.006, 0.023]	0.0011	OLS: inflammaging_score (raw senescence terms)	nan
CD8 CCR7+ (%)	−0.004 [−0.015, 0.006]	0.4102	OLS: inflammaging_score (raw senescence terms)	nan
Age (years)	−0.023 [−0.035, −0.012]	0.0001	OLS: inflammaging_score (raw senescence terms)	nan
CMV IgG positive	−0.140 [−0.276, −0.004]	0.0436	OLS: inflammaging_score (raw senescence terms)	nan
log1p(hs-CRP)	nan	<0.0001	Logit: severe periodontitis (raw inflammaging terms)	28.08 [5.43, 145.12]
log1p(IL-6)	nan	<0.0001	Logit: severe periodontitis (raw inflammaging terms)	16.90 [4.22, 67.79]
log1p(TNF-α)	nan	0.0011	Logit: severe periodontitis (raw inflammaging terms)	14.42 [2.91, 71.44]
log(RANKL/OPG)	nan	<0.0001	Logit: severe periodontitis (raw inflammaging terms)	13.39 [6.14, 29.22]
Immunosenescence score (z)	nan	0.6290	Logit: severe periodontitis (raw inflammaging terms)	1.24 [0.52, 2.93]
Age (years)	nan	0.1731	Logit: severe periodontitis (raw inflammaging terms)	0.94 [0.87, 1.03]
CMV IgG positive	nan	0.7440	Logit: severe periodontitis (raw inflammaging terms)	0.83 [0.27, 2.55]
Low BMD	nan	<0.0001	Logit: severe periodontitis (raw inflammaging terms)	0.09 [0.03, 0.28]

OLS coefficients (HC3) and OR (95% CI) for the logistic model.
