# Supplementary material for: Osteoimmunologic and Immune-Aging Signatures in Postmenopausal Women with Periodontitis and Low Bone Mineral Density: A Cross-Sectional Study
Source: Diagnostics (Basel). 2026 Feb 27;16(5):708. doi: 10.3390/diagnostics16050708 (PMC12984706; doi:10.3390/diagnostics16050708)
Supplement: Supplementary file 1 [file diagnostics-16-00708-s001.zip › diagnostics-4157232-supplementary.pdf]

Supplementary Material

Table S1. Sensitivity analysis separating osteopenia and osteoporosis within the low-BMD stratum

Table S1a. In the primary analyses, osteopenia and osteoporosis were combined into a single low-BMD category (DXA T-score < −1.0) to preserve cell sizes for the prespecified 2×2 factorial models. As a sensitivity analysis, we examined whether results differed materially when separating osteopenia from osteoporosis among participants with low BMD. Descriptive statistics within low BMD (T-score < −1.0).

BMD category	n	Age, mean (SD)	Inflammaging z, mean (SD)	log(RANKL/OPG), mean (SD)	RANKL/OPG, median (IQR)
Osteopenia	95	67.6 (5.4)	0.42 (0.58)	0.93 (0.63)	2.39 (1.98)
Osteoporosis	45	68.2 (4.0)	0.17 (0.56)	1.08 (0.76)	2.75 (3.83)

Table S1b. Adjusted models (restricted to low BMD) used robust linear regression (HC3) with outcomes (i) inflammaging composite z-score and (ii) log(RANKL/OPG), including an indicator for osteoporosis (vs osteopenia) and adjusting for age, residence (rural vs urban), CMV serostatus, and periodontal status (severe vs no/mild).

Outcome	Contrast	β (95% CI)	*p*-value
Inflammaging composite (z)	Osteoporosis vs osteopenia	-0.261 (-0.438 to -0.084)	0.0038
log(RANKL/OPG)	Osteoporosis vs osteopenia	0.107 (-0.121 to 0.335)	0.3573

The primary inference that periodontal status is associated with higher inflammaging and higher log(RANKL/OPG) remained unchanged. Within the low-BMD stratum, osteoporosis (vs osteopenia) showed a modest inverse association with inflammaging after adjustment, while it was not associated with log(RANKL/OPG). These estimates should be interpreted as associative and model-conditioned in this cross-sectional dataset.

Table S2. Composite-score diagnostics (internal consistency and collinearity)

Table S2a. The composite scores for inflammation and immunosenescence were prespecified as biologically motivated summary indices. For transparency, internal consistency was assessed using Cronbach's alpha (α) on the standardized components. Collinearity among components was evaluated using variance inflation factors (VIFs) from linear models with an intercept.

Composite score	Components	Cronbach's α
Inflammaging	log hs-CRP, log IL-6, log TNF-α (z-scored)	0.51

Immunosenescence	CD28−, CD57+, KLRG1+, PD-1+, CD27− (z-scored) with CCR7 and CD45RA reverse-coded	0.78

Table S2b. Component-level VIFs (standardized components)

Inflammaging components:

Component	VIF
hsCRP_mg_L	1.14
IL6_pg_mL	1.13
TNFalpha_pg_mL	1.10

Immunosenescence components:

Component	VIF
CD28neg_pct_CD8	3.50
CD57_pos_pct_CD8	2.65
KLRG1_pos_pct_CD8	2.46
PD1_pos_pct_CD8	1.91
CD27neg_pct_CD8	2.67
CCR7_pos_pct_CD8	2.27
CD45RA_pos_pct_CD8	1.44

Cronbach's α for inflammaging was modest, consistent with the intent to summarize partially distinct inflammatory pathways rather than a unidimensional psychometric scale. Immunosenescence α was higher, consistent with clustering of late-differentiation phenotypes. VIFs were within acceptable ranges, indicating no problematic multicollinearity among components.

Table S3. Effect sizes for 2×2 factorial ANOVA models (partial η^2). Partial η^2 values were computed from type II sums of squares as SS_effect / (SS_effect + SS_error).

Outcome	Effect	F	*p*-value	partial η^2
Inflammaging (z)	Periodontal status	189.23	<0.0001	0.407
Inflammaging (z)	BMD status	139.95	<0.0001	0.336
Inflammaging (z)	Interaction	16.13	<0.0001	0.055
Immunosenescence (z)	Periodontal status	20.15	<0.0001	0.068
Immunosenescence (z)	BMD status	39.65	<0.0001	0.126
Immunosenescence (z)	Interaction	0.38	0.5392	0.001
log(RANKL/OPG)	Periodontal status	150.87	<0.0001	0.353
log(RANKL/OPG)	BMD status	15.43	0.0001	0.053
log(RANKL/OPG)	Interaction	5.37	0.0212	0.019

Table S4. Multivariable linear models with standardized coefficients. Models were estimated using OLS with HC3 robust standard errors. Standardized β were computed as β × SD(X)/SD(Y).

Outcome	Predictor	β	CI_low	CI_high	*p*-value	Standardized β
Inflammaging (z)	CMV_IgG_positive[T.True]	-0.173	-0.319	-0.027	0.0199	-0.106
Inflammaging (z)	severe_periodontitis	0.643	0.500	0.786	<0.0001	0.452
Inflammaging (z)	low_bmd	0.664	0.537	0.792	<0.0001	0.467
Inflammaging (z)	immunosenescence_z	0.294	0.172	0.417	<0.0001	0.270
Inflammaging (z)	log_ranklopg	0.033	-0.060	0.127	0.4817	0.034
Inflammaging (z)	age_years	-0.024	-0.036	-0.012	<0.0001	-0.195
Inflammaging (z)	rural	0.040	-0.072	0.152	0.4820	0.028
Immunosenescence (z)	CMV_IgG_positive[T.True]	0.618	0.482	0.753	<0.0001	0.413
Immunosenescence (z)	severe_periodontitis	0.083	-0.073	0.239	0.2996	0.063
Immunosenescence (z)	low_bmd	0.037	-0.114	0.189	0.6313	0.028
Immunosenescence (z)	inflammaging_z	0.305	0.195	0.415	<0.0001	0.332
Immunosenescence (z)	log_ranklopg	0.041	-0.058	0.140	0.4195	0.045
Immunosenescence (z)	age_years	0.037	0.026	0.048	<0.0001	0.331
Immunosenescence (z)	rural	-0.041	-0.154	0.073	0.4859	-0.031
log(RANKL/OPG)	CMV_IgG_positive[T.True]	-0.049	-0.227	0.129	0.5917	-0.029
log(RANKL/OPG)	severe_periodontitis	0.797	0.621	0.972	<0.0001	0.549
log(RANKL/OPG)	low_bmd	0.115	-0.085	0.315	0.2589	0.079
log(RANKL/OPG)	inflammaging_z	0.051	-0.092	0.193	0.4846	0.050

log(RANKL/OPG)	immunosenescence_z	0.060	-0.082	0.202	0.4089	0.054
log(RANKL/OPG)	age_years	0.015	0.001	0.029	0.0315	0.121
log(RANKL/OPG)	rural	0.054	-0.084	0.193	0.4405	0.037

Abbreviations: BMD, bone mineral density; CMV, cytomegalovirus; CI, confidence interval.

Figure S1. Interaction plots for key 2×2 factorial ANOVA findings. Cell means (±95% CI) are shown for the factorial design of periodontal status × BMD status. Interaction magnitude is summarized as a difference-in-differences (DiD): (Severe_low − Severe_normal) − (No/mild_low − No/mild_normal).

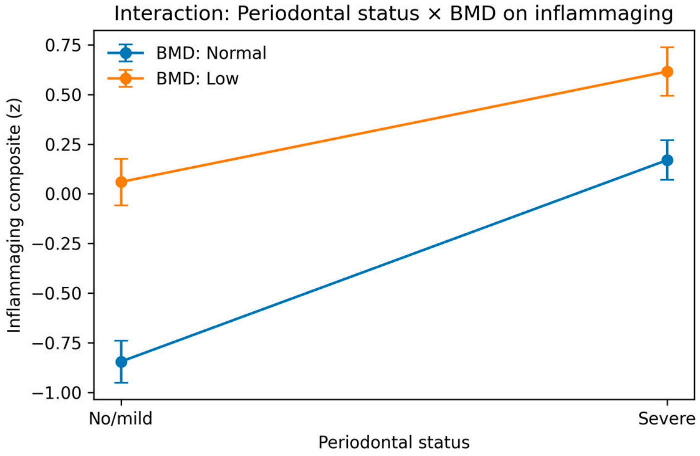

Panel A. Inflammaging composite (z-score). Interaction term: $F(1,276) = 16.13$, $p = 7.618e\text{-}05$; partial $\eta^2 = 0.055$. DiD = -0.458 z-units.

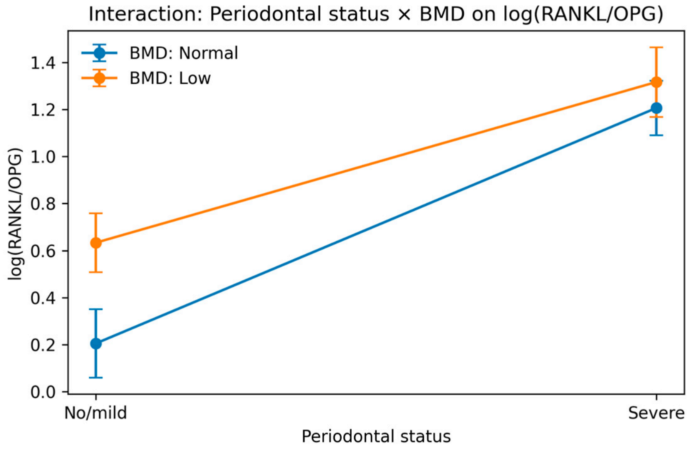

Panel B. log(RANKL/OPG). Interaction term: $F(1,276) = 5.37$, $p = 0.02116$; partial $\eta^2 = 0.019$. DiD = -0.318 log-units.

Table S5. Examiner calibration for periodontal measurements. Examiner calibration was performed on a training set of 12 postmenopausal patients not included in the main study. Full-mouth PD and CAL were recorded at six sites per tooth and repeated after 48–72 h under identical conditions. Intra-examiner reproducibility was quantified using a two-way mixed-effects intraclass correlation coefficient (ICC, absolute agreement).

Measurement	ICC (absolute agreement)	95% CI	Target ICC
Probing depth (PD)	0.88	0.83–0.92	≥0.80
Clinical attachment level (CAL)	0.84	0.78–0.89	≥0.80

These ICCs indicate high intra-examiner reliability for periodontal measurements and meet the prespecified reproducibility target.

Table S6. Effect sizes for factorial ANOVA main effects and interactions

Outcome	Effect	F (df=1,276)	p-value	partial η^2
Inflammaging composite (z)	Periodontal status	189.23	3.817e-33	0.407
Inflammaging composite (z)	BMD status	139.95	2.148e-26	0.336
Inflammaging composite (z)	Interaction (Perio×BMD)	16.13	7.618e-05	0.055
log(RANKL/OPG)	Periodontal status	150.87	5.864e-28	0.353
log(RANKL/OPG)	BMD status	15.43	0.000108	0.053
log(RANKL/OPG)	Interaction (Perio×BMD)	5.37	0.02116	0.019
